# Freezing mediastinal lymph node: first case of mediastinal cryobiopsy guided by EBUS in Brazil

**DOI:** 10.36416/1806-3756/e20230315

**Published:** 2024-04-08

**Authors:** João Pedro Steinhauser Motta, Amir Szklo, Bianca Peixoto Pinheiro Lucena, Marcos de Carvalho Bethlem, Leonardo Hoehl Carneiro

**Affiliations:** 1. Instituto de Doenças do Tórax, Universidade Federal do Rio de Janeiro, Rio de Janeiro (RJ), Brasil.

## TO THE EDITOR,

Endobronchial ultrasound-guided transbronchial needle aspiration (EBUS-TBNA) is currently considered the method of choice for the invasive mediastinal staging of lung cancer. The samples obtained through EBUS-TBNA have proven suitable for tumor subtyping, immunohistochemistry, and molecular analysis. Transbronchial cryobiopsy is a technique used in the diagnosis of interstitial lung diseases that enables obtaining more tissue and samples with preserved architecture. A combination of EBUS and cryobiopsy, known as transbronchial mediastinal cryobiopsy guided by EBUS (EBUS-TBMC), is being developed as a novel mediastinal sampling strategy.^1^ EBUS-TBMC can be considered a complementary method to EBUS-TBNA in cases where material is difficult to obtain through a cytological needle (metastatic lymph nodes after chemotherapy, immunotherapy, or radiotherapy) and in cases of difficult diagnosis, such as lymphoproliferative diseases and benign infectious and inflammatory lesions, in which histological material offers advantages over cytological samples.^2^ ^,^ ^3^ In the present letter, we describe the first case of EBUS-TBMC performed in Brazil.

**Figure 1:**
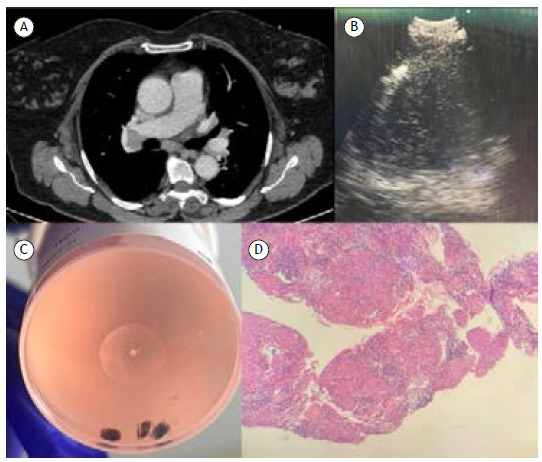
(A) Chest tomography with infracarinal and right hilar lymph node enlargement. (B) EBUS image showing the cryoprobe inside the lymph node. (C) EBUS-TBMC samples. (D) Histopathological image of the granuloma.

We present a case of a 50-year-old female patient, a former smoker, who presented with mediastinal lymph node enlargement, periodic fever, and cough over the past three months. We chose to perform EBUS-TBMC due to the need for a differential diagnosis between lymphoproliferative disease, sarcoidosis, granulomatous infection, or lung cancer. After providing informed consent, EBUS-TBMC was performed. The procedure was conducted on an outpatient basis, under general anesthesia, using an orotracheal tube and endobronchial blocker. An infracarinal lymph node measuring 15 mm was identified. After four passes of TBNA with a 22-gauge needle, a 1.1 cryoprobe was introduced through the working channel of the EBUS bronchoscope. The cryoprobe was then advanced into the target lesion through the bronchial-wall orifice created by the needle. Upon confirming the cryoprobe’s position inside the lymph node through ultrasound visualization, transbronchial mediastinal cryobiopsy (EBUS-TBMC) (Figure 1) was performed by cooling down for 3 seconds, followed by en bloc endoscopic removal. A total of 4 cryobiopsies were carried out, with subsequent sample fixation in formalin. No significant bleeding, pneumothorax, or other clinical complications were observed. Histopathological analyses revealed chronic granulomatous lymphadenitis without necrosis, with negative staining for microorganisms, suggesting a diagnosis of sarcoidosis. 

We aimed to present the first EBUS-TBMC performed in Brazil, an innovative biopsy technique that allows for the retrieval of larger mediastinal tissue samples with preserved architecture. Traditionally, EBUS is performed by needle aspiration and has shown good performance in diagnosing and staging lung cancer. Combining transbronchial cryobiopsy with linear EBUS provides histopathological samples with preserved architecture, which can be helpful in cases where histological material has advantages over cytological samples.

The primary technical challenge encountered was the perforation of the bronchial wall and the lymph node capsule with the cryoprobe, given its lack of sharpness. We utilized a 22-gauge needle to perform the TBNA, and introduced the cryoprobe through the same puncture site. Some authors have suggested alternative techniques, such as using a 21G needle to create a larger puncture hole or a knife with electrocoagulation.^2^ Another method described in the literature involves making multiple puncture holes with the TBNA needle to break the capsule and facilitate the passage of the cryoprobe.^4^ Following the initial cryobiopsy, the already created pathway can be used for subsequent biopsies without major difficulties. The cryosamples analyzed by the pathologist were compared with the TBNA samples. Although both sample types were adequate for establishing the diagnosis of sarcoidosis, the EBUS-TBMC sample allowed for the visualization of the preserved architecture of the granuloma and the delineation of how the histiocytes were organized. 

In this report, we share our pioneering experience in Brazil with this promising new biopsy technique. The procedure was uneventful, and the histopathological result played a decisive role in establishing the diagnosis of the patient in question. We will soon draft an original article describing the EBUS-TBMC case series currently under study at our center.
